# Integrating preconception carrier screening into public health: lessons learned from a pilot implementation study

**DOI:** 10.1007/s10815-026-03871-9

**Published:** 2026-05-04

**Authors:** Aina Marsal-Olivan, Clara Serra-Juhe, Alicia Artigas-Baleri, Sara Bernal, Ivon Cusco, Anna Abulí, Carlos Brotons, Benjamín Rodríguez-Santiago, Jordi Surralles

**Affiliations:** 1 Institut de Recerca Sant Pau, IR SANT PAU, Barcelona, Spain; 2 Departament de Genètica i Microbiologia, Autonomous University of Barcelona, Cerdanyola del Vallès, Spain; 3 Servei de Genètica, Hospital de Sant Pau, Barcelona, Spain; 4 Centro de Investigación Biomédica en Red de Enfermedades Raras, Madrid, Spain; 5 Genetics Unit, Hospital del Mar Medical Research Institute, Barcelona, Spain; 6 Sardenya Primary Health Care Centre, Barcelona, Spain; 7 Unitat Mixta de Recerca en Medicina Genòmica, UAB-IR SANT PAU, Barcelona, Spain; 8 Unitat Docent Multiprofessional d’Atenció Familiar i Comunitària Aceba, Barcelona, Spain

**Keywords:** Carrier screening, Massively parallel sequencing, Preconception screening, Reproductive decision-making, Public health implementation

## Abstract

**Purpose:**

Carrier screening identifies individuals at risk of transmitting autosomal recessive or X-linked recessive conditions, supporting informed reproductive decisions. Despite international recommendations for universal carrier screening, integration into public healthcare systems remains limited. This study evaluated the feasibility of implementing a carrier screening program in Spain’s public health system.

**Methods:**

Non-pregnant women aged 18 to 38 with reproductive intent were recruited through text messages sent from a primary care center, along with their partners. A sequential screening approach was used: women underwent a 351-gene massively parallel sequencing panel and complementary tests, while male partners were tested only if their partner carried autosomal recessive variants. Specific genetic counselling was provided, and emotional responses were assessed through pre- and post-test questionnaires.

**Results:**

Of 518 candidates contacted, 400 (77%) responded positively and 152 couples (50.8% of those eligible) enrolled. Among 218 individuals screened, 62% (135) carried at least one pathogenic variant. Six carrier couples (4%) were identified as being at reproductive risk of non-syndromic hearing loss (*GJB2*, 2 couples), cystic fibrosis (*CFTR*), Stargardt disease and retinitis pigmentosa (*ABCA4*), Smith-Lemli-Opitz syndrome (*DHCR7*), and Fabry disease (*GLA*). Of four couples followed post-disclosure, 75% opted for in vitro fertilization with preimplantation genetic testing or prenatal diagnosis. Prevention of severe genetic conditions was the most frequently reported motivation for participation.

**Conclusions:**

This study proves the feasibility and clinical utility of carrier screening within a public healthcare system. Our findings demonstrate high participation rates, clinical relevance through the identification of 4% of carrier couples, and strong motivation among participants to prevent the transmission of severe genetic conditions.

**Supplementary Information:**

The online version contains supplementary material available at 10.1007/s10815-026-03871-9.

## Introduction

Mendelian diseases, though individually rare, collectively represent a significant burden on both families and public health, contributing to more than 20% of infant mortality and 10% of pediatric hospitalizations [1]. More than 2000 of these disorders follow autosomal recessive (AR) or X-linked recessive (XLR) inheritance patterns, affecting approximately 1 in 300 births [2].

Carrier screening (CS) is a genetic testing approach designed to identify individuals with a pathogenic variant associated with a recessive disorder. While symptoms of the condition are not typically expressed, recent evidence suggests that some heterozygous carriers, particularly those with variants in constrained genes or genes associated with neurodevelopmental disorders, may exhibit subtle phenotypic effects, such as lower educational achievement or reduced reproductive fitness [3]. Nevertheless, the main concern remains the potential to transmit the genetic variant to their children, as couples in which both partners are carriers of the same AR condition or the woman is a carrier of an XLR condition have a 25% risk of having an affected child in each pregnancy.

Over the years, several studies have estimated the average burden of recessive pathogenic variants per individual. Early research suggested that individuals carry 3–5 pathogenic recessive variants [4], but more recent large-scale screening programs have estimated an average of 2 pathogenic variants per person [5, 6]. The frequency of carrier couples, those at risk of passing on a genetic disorder, varies widely, with large-scale screening programs estimating that around 2% of couples fall into this category [6–12].

Carrier testing can be offered as an occasional test, as part of a population-based screening program, or targeted at high-risk populations based on ancestry or family history [13]. It can be provided to individuals or couples, with testing performed either simultaneously or sequentially, where the second partner is tested only if the first tests positive [13]. Additionally, results can be communicated individually, disclosing each person’s results separately, or on a couple-based approach, where only couples in which both partners are carriers are notified with a positive result [13].

The implementation of CS has significant implications for reproductive decision-making [13]. While CS can be offered preconceptionally, prenatally, or premaritally, the preconception approach maximizes reproductive choices. By identifying carrier couples before pregnancy, screening enables individuals to consider different reproductive options, including preimplantation genetic testing (PGT), prenatal diagnosis with the possibility of termination of pregnancy (TOP), gamete donation, adoption, or preparing for the birth of an affected child [13, 14]. Despite its potential benefits and the widespread recognition of its value, reported CS uptake remains inconsistent, with participation rates ranging from 8 to 77% depending on factors such as timing, access, and individual perception of risk (Supplementary Table 1). Sparing a child from a life with a severe genetic condition is reported to be the most important reason for accepting carrier screening [15, 16].

Professional organizations, including the American College of Medical Genetics and Genomics (ACMG), the American College of Obstetricians and Gynecologists (ACOG), and the European Society of Human Genetics (ESHG), provide guidelines on CS implementation [13, 17–21]. While there is consensus that CS should be offered to all individuals regardless of ancestry, differences remain in the criteria used to select conditions for screening. Some organizations support limited panels that focus on prevalent, severe, early-onset diseases with clear genotype–phenotype correlations [13, 22], while others consider expanded panels that include a broader range of conditions [18, 20]. However, there is broad agreement that CS panels should pursue a balance between comprehensive genetic condition coverage and clinical relevance, prioritizing clinical validity, utility, and actionability while avoiding unnecessary testing and supporting informed reproductive decision-making [21–23].

The implementation of CS into routine practice is challenging. Concerns related to anxiety, stigmatization, and other ethical considerations have been raised regarding expanded screening panels, which include a broad range of conditions beyond those traditionally recommended for screening [16, 24]. Therefore, carrier screening programs vary significantly across the world [25]. In Spain and many other European countries with universal public health care system, CS is widely available and implemented in the private sector, particularly within assisted reproduction clinics, but it has not yet been integrated into the public healthcare system [26]. This creates inequity in access, limiting the availability of genetic screening for the general population. In response to these challenges, Spanish scientific societies of reference have published joint guidelines outlining recommendations for CS implementation, including criteria for panel design and clinical applicability [22]. Given international recommendations and the growing interest in CS, evaluating the feasibility of incorporating the test into public healthcare is crucial. This study presents the first implementation study for CS within Spain’s public health system, assessing its feasibility, clinical utility, acceptance, perception of risk, and potential impact on reproductive decision-making.

## Materials and methods

### Carrier screening strategy

**Fig. 1:**
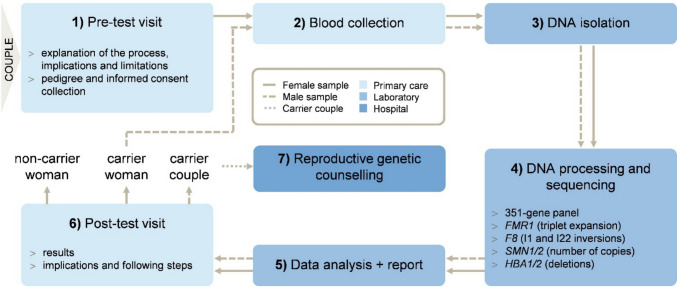
Overview of the carrier screening process. Steps conducted in different centers are highlighted in different intensities of color. Female sample steps are indicated by continuous lines, male samples by dashed lines, and carrier couples by dotted lines

A sequential screening approach was adopted to enhance efficiency and minimize unnecessary testing. Under this strategy, women were screened first to identify carriers of AR and XLR conditions. Male partners were only tested if their female partner had been identified as a carrier of an AR condition (Fig. 1).

### Recruitment and sample collection and processing

The study was conducted in close coordination between Hospital de la Santa Creu i Sant Pau (HSCSP), Sant Pau Research Institute and Sardenya Primary Health Care Centre (Barcelona, Spain), between December 2023 and March 2024. Women aged 18 to 38 registered at the primary health care center received an SMS invitation to participate in a free CS program, with the following message: “*Do you want to know your risk of having a child with a genetic condition? Answer YES or NO to take part in a new study being conducted at the center*.” Those who responded positively were contacted by telephone to confirm eligibility. Eligible participants had to be non-pregnant at the time of recruitment, have a male reproductive partner willing to participate in the screening process, and express current or future reproductive desire. Eligible couples were required to attend a pre-test genetic counselling session, where they received comprehensive information about the benefits, limitations, and reproductive implications of carrier screening before providing informed consent. Individuals with known genetic diagnoses were not excluded from participation, as they could still be carriers of additional recessive conditions unrelated to their primary diagnosis. However, none of the included participants had a diagnosis of a recessive disorder.

Peripheral blood samples were collected in EDTA tubes. DNA was isolated using the Wizard Genomic DNA Purification Kit (Promega; Madison, Wisconsin, USA).

### Massively parallel sequencing analysis

A massively parallel sequencing gene panel targeting 351 genes associated with 451 genetic disorders was newly designed and validated (Supplementary Table 2). Gene selection was based on criteria ensuring clinical relevance, including association with conditions with early onset that impact quality of life due to cognitive or sensory impairment, other severe phenotypes, and/or reduced life expectancy, aligning with the recommendations published by scientific societies of reference [13, 17–22]. Additional selection factors included strong gene-phenotype associations, high carrier rate in the population of reference, inclusion in the public biochemical neonatal screening program, and the potential for prenatal diagnosis, PGT, or early intervention. Certain genes associated with milder or later-onset phenotypes were retained in the panel despite not strictly meeting the initial core selection criteria, as the panel composition was based on a pre-existing expanded carrier screening tests.

DNA libraries were prepared using a capture‑based target enrichment approach with the KAPA HyperPlus Kit (Roche; Basel, Switzerland). Libraries were then pooled in equimolar amounts and hybridized to KAPA HyperCapture HyperChoice Probes, targeting a custom gene panel with a total target size of 0.86 Mb. Sequencing was performed on NextSeq500 (2 × 150 bp) and NextSeq1000 (2 × 100 bp) instruments (Illumina; San Diego, CA, USA) at the HSCSP sequencing facility. Sequencing quality was assessed using Picard tools. The average target coverage achieved was 590 ×, with 99.8% of bases covered at ≥ 20 ×, ensuring high sensitivity for SNV, indel, and CNV detection. FASTQ files were generated after demultiplexing and quality‑controlled with FastQC. Adapter trimming was performed using Trimmomatic, and reads were aligned to the human reference genome (GRCh37) using BWA‑MEM v0.7.17 [27]. Variant calling for SNVs and small indels was carried out with GATK v4.2 [28], and variants were filtered and annotated using ANNOVAR [29] together with custom annotation scripts. CNV detection was performed on coverage‑normalized BAM files using ExomeDepth [30], with genomic interval handling implemented via GenomicRanges and IRanges (Bioconductor; Seattle, WA, USA). All candidate SNVs, indels, and CNVs were manually reviewed in Integrative Genomics Viewer (IGV) [31].

Variants were classified according to ACMG/AMP guidelines [32], with pathogenic or likely pathogenic variants reported in detail. Variants of uncertain significance (VUS) were excluded from the clinical report.

### Supplementary techniques

In addition to the massively parallel sequencing-based analysis, supplementary testing was conducted to detect specific pathogenic variants not covered by standard short-read sequencing analysis. The AmplideX® PCR/CE SMN1/2 Plus Kit (Asuragen; Austin, TX, USA) was used to assess spinal muscular atrophy carrier status by determining the number of copies of the *SMN1*/*SMN2* genes. The SALSA® MLPA® Probemix P140 HBA kit (MRC-Holland; Amsterdam, The Netherlands) was employed for α-thalassemia to identify deletions in the *HBA1*/*HBA2* genes. Inverse PCR was performed to detect intron 1 and 22 inversions in the *F8* gene, which is associated with hemophilia A. Lastly, triplet-primed PCR (TP-PCR) and/or the AmplideX® FMR1 PCR Kit (Asuragen; Austin, TX, USA) were used to detect CGG repeat expansions in the *FMR1* gene, related to fragile X syndrome.

### Quality control and sample validation

The analytical performance of the panel was assessed through two complementary validation studies. First, retrospective validation was performed using 48 DNA samples from the HSCSP, each harboring previously confirmed pathogenic variants in genes represented in the panel. Second, a reference-based validation was conducted using NA24385 DNA (Coriell Institute), a well-characterized sample from the Genome in a Bottle (GIAB) consortium (available at: https://ftp-trace.ncbi.nlm.nih.gov/). Sensitivity, specificity, and technical error rates were determined by comparing SNV and indel calls to GIAB’s high-confidence variant set. The panel achieved 100% sensitivity and 99.9997% specificity, with all expected known variants detected, excluding those inaccessible to short-read sequencing technologies (data not shown).

To ensure sample integrity and sequencing accuracy, a 14-marker SNP-based MLPA assay was conducted. Results were cross-referenced with massively parallel sequencing-derived SNP genotypes to verify sample identity and eliminate potential errors related to sample mislabeling, mix-up, or contamination.

### Genetic counselling visits and clinical management

The genetic counselling protocol consisted of pre-test and post-test counselling sessions to ensure participants received comprehensive information and support throughout the screening process. During the pre-test counselling session, which lasted approximately 45 min, all participants were provided with a detailed explanation of the carrier screening process, including its scope, limitations, and potential reproductive implications. Additionally, a three-generation pedigree was collected to assess family history of genetic conditions, and participants were required to review and sign informed consent before proceeding with the test.

Following genetic testing, two post-test counselling sessions were conducted according to the sequential screening strategy. The first post-test visit focused on the female participants’ results, with results delivered in person for carriers and via telephone for non-carriers. If a woman was identified as a carrier of an AR condition, her male partner was offered testing. In cases where a woman was a carrier of an X-linked condition, reproductive options were discussed in detail. The second post-test visit involved the male partner’s results and, when applicable, a discussion of the couple’s reproductive risk. If the male partner tested negative, results were communicated via telephone. In contrast, if both partners were carriers of the same autosomal recessive condition, the couple was offered an in-person consultation to discuss their reproductive options in detail. Post‑test counselling sessions required around 30 min for carriers and a 10‑min telephone call for non‑carriers.

Carrier couples were subsequently referred to the reproductive genetic counselling for further specialized support and management, ensuring they received appropriate guidance regarding family planning and available reproductive alternatives.

### Survey evaluation

Participants completed structured online surveys before and after genetic testing. Both surveys collected demographic information, assessed scientific knowledge [33, 34], measured perceived personal control (PPC) with a validated 6-item questionnaire [35, 36], and evaluated anxiety using a 7-item adapted of the State-Trait Anxiety Inventory (STAI) [37]. The pre-test survey also explored motivations and expectations, while the post-test survey assessed satisfaction [38]. Both surveys were distributed electronically through the REDCap platform, ensuring standardized, confidential data collection [39].

### Statistical analysis

All statistical analyses were performed using Jamovi (version 2.3.28). The Wilcoxon signed-rank test was applied to compare two related samples. Spearman’s rank correlation coefficient was used to assess associations between continuous, non-normally distributed variables. To compare differences across multiple independent groups, the Kruskal–Wallis test, a non-parametric alternative to one-way ANOVA, was employed. A *p*-value of ≤ 0.05 was considered statistically significant for all analyses.

## Results

### Study population

**Fig. 2:**
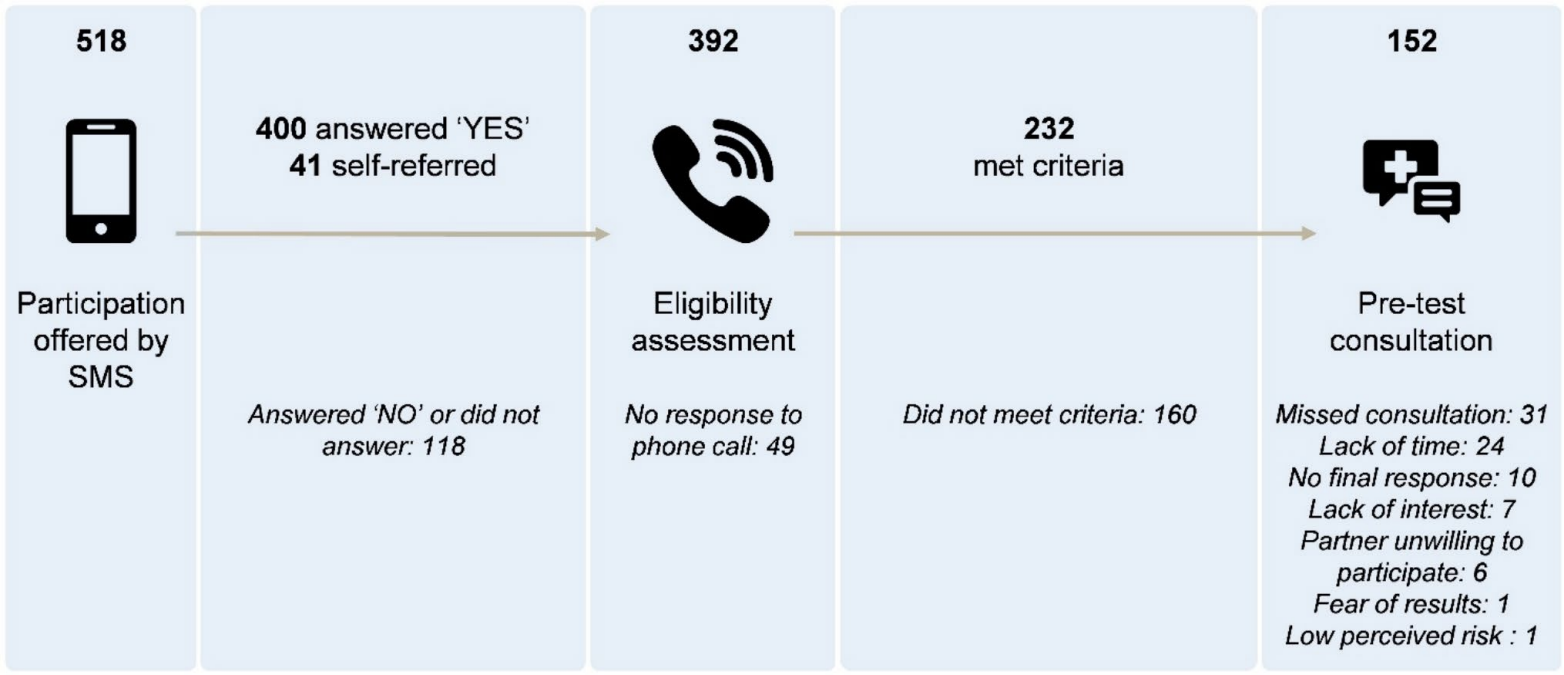
Flowchart illustrating participant recruitment and enrolment in the CS program. Reasons for non-participation are detailed in the lower section of the chart. The enrolment process focused on female partners

A total of 518 women aged 18 to 38 were contacted via SMS and invited to participate in the CS program, of whom 400 (77%) accepted. An additional 41 self-referred after learning about the study through various sources. Of the 441 interested participants, 392 were successfully contacted by telephone to receive a general overview of the study and to assess their eligibility based on the inclusion criteria; the remaining 49 did not respond to the call. Among those contacted, 232 met the eligibility requirements. However, 80 ultimately did not enroll for various reasons: failure to attend the consultation (*n* = 31), lack of time or availability for genetic counselling (*n* = 24), no further response after the initial call (*n* = 10), loss of interest (*n* = 7), partner unwilling to participate (*n* = 6), concern about the results (*n* = 1), and perceived low risk of having a child with a genetic condition (*n* = 1) (Fig. 2). The final study cohort consisted of 152 couples (152 women and 66 male partners of women identified as carriers), two of whom were second-cousins. Demographic characteristics are displayed in Supplementary Table 3. Most participants were aged 26–38 years (89%) and had at least a Bachelor’s degree (84%).

### Screening outcomes

**Fig. 3:**
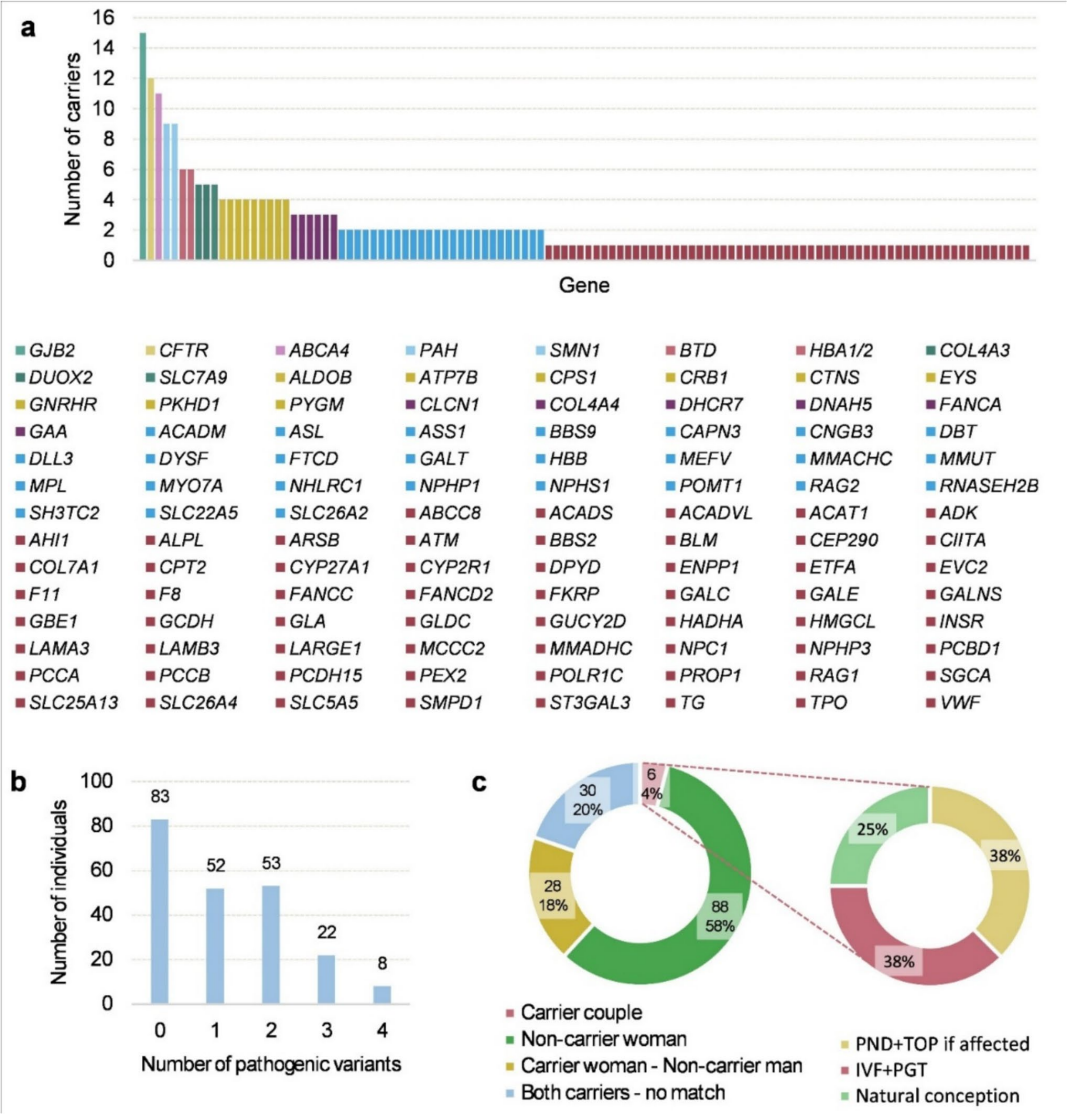
Carrier screening outcomes. **a** Distribution of genes with pathogenic variants identified in the study. The bar plot displays the number of carriers (y-axis) for each gene (x-axis). Genes are ordered from highest to lowest number of carriers, with alphabetical ordering applied for genes with the same number of carriers. **b** Distribution of the number of pathogenic or likely pathogenic variants identified per individual. The bar plot shows the frequency of individuals (y-axis) carrying a specific count of pathogenic variants (x-axis). **c** Carrier screening outcomes in couples. (Left) Couples’ carrier status. (Right) Reproductive options selected by four carrier couples for ongoing and future pregnancies. *IVF* in vitro fertilization, *PGT* preimplantation genetic testing, *PND* prenatal diagnosis, *TOP* termination of pregnancy

Overall, 250 pathogenic or likely pathogenic variants were found in 112 genes. Sixty-two percent of the individuals analyzed were identified as carriers of at least one recessive genetic condition (135/218). The mean number of pathogenic variants identified per individual was 1.17, increasing to 1.9 among the carrier population and excluding non-carriers. The number of pathogenic variants per person ranged from 0 to 4 (Fig. 3b). The genes with the highest carrier frequencies were *GJB2* (15 carriers), *CFTR* (12 carriers), *ABCA4* (11 carriers), *PAH* (8 carriers), and *SMN1* (8 carriers) (Fig. 3a).

Among the 152 couples who underwent CS, 6 (4%) were identified as having an increased risk of having offspring affected by a genetic condition. In five cases, both partners were carriers of the same AR condition (nonsyndromic hearing loss, *GJB2* (2 couples); cystic fibrosis, *CFTR*; Stargardt disease and retinitis pigmentosa, *ABCA4*; and Smith-Lemli-Opitz syndrome, *DHCR7*), while in one case, the woman was a carrier of an XLR condition (Fabry disease; *GLA*) (Supplementary Table 4). Four of the six carrier couples completed a follow-up interview approximately 1 year after receiving their results. One couple declined participation, and another was not yet considering pregnancy at the time. Participants described an initial emotional response characterized by shock, fear, and uncertainty. Over time, these feelings diminished as couples accessed further information and support, resulting in increased reassurance and a sense of empowerment. Overall, the couples expressed appreciation for having received this information early in their reproductive decision-making process.

Most couples acknowledged that their carrier status influenced their reproductive choices. Two couples—carriers of *CFTR* and *ABCA4*—were in early pregnancy at the time of result disclosure and opted for prenatal diagnosis. Both indicated they would have considered termination if the fetus had been affected; in both cases, pregnancies continued with unaffected babies. They also stated that, if they had not already been pregnant or for future pregnancies, they would pursue IVF with PGT to avoid transmitting the condition to their offspring. Among the two non-pregnant couples, one (*DHCR7* carriers) underwent two cycles of IVF with PGT, while the other (*GJB2* carriers) pursued natural conception due to a low ovarian reserve. The latter couple also noted that they would not have considered pregnancy termination, even if prenatal testing revealed an affected fetus.

### Survey outcomes

Survey completion rates varied between the pre-test and post-test phases, with higher participation before testing. A total of 74% of participants completed the pre-test survey, with 85% of women and 49% of men responding. In contrast, response rates declined significantly for the post-test survey, with only 23% of participants completing it (24% of women and 22% of men) (Supplementary Table 3). Most participants reported a decrease in anxiety when comparing pre-test and post-test results (53%), without statistically significant evidence (Supplementary figure a–c). Statistical correlations were identified for two factors. STAI and PPC were significantly negatively correlated, indicating that higher anxiety levels were associated with lower perceived control over the situation (Supplementary figure d). Scientific knowledge was significantly associated with the level of education (Supplementary figure e).

The pre-test survey also assessed motivations for enrolling in the carrier screening program, allowing participants to select multiple reasons. The predominant motivation, cited by 73% of respondents (116/160), was to prevent offspring from inheriting severe genetic diseases. Emotional and risk-based factors also influenced test uptake, including fear of regret of declining participation (22%) and a high perceived likelihood of being a carrier (15%). Additionally, 14% (23/160) reported a family history of hereditary disorders. However, as the surveys were anonymous, the specific referred conditions could not be determined, and this subgroup was therefore too small and too heterogeneous to support meaningful statistical comparisons. Smaller proportions participated due to a wish to prepare for the possibility of having an affected child (13%), recommendations from another person (9%), or partner’s request (8%).

## Discussion

This study provides a proof of concept for CS implementation pipeline within Spain’s public healthcare system, offering insights into its feasibility, clinical utility, and psychosocial impact. The study demonstrated a strong interest in reproductive CS within the general population, with 77% of invited women responding positively to the SMS invitation. To our knowledge, this represents one of the highest levels of reported interest in CS to date (Supplementary Table 1). Previous research has highlighted a discrepancy between hypothetical interest (ranging from 32 to 80%) and actual uptake (typically 8–50%). In the present study, while 77% of invitees expressed interest and 66% of eligible participants undergone testing, the final uptake rate was 50.8% of those initially contacted. This figure is comparable to uptake rates reported in the Australian free-of-charge screening initiative; Kirk et al. [6] found that an estimated 45.9% of those invited proceeded with reproductive genetic carrier screening [6].

Logistical barriers, including missed consultations and time constraints, reduced the final cohort to 152 women, along with the partners of those identified as carriers. Notably, only one participant declined due to anxiety about the results, which contrasts with previous studies where fear was one of the dominant barriers [16, 24]. This suggests that the pre-test phone call may have reduced concerns in this cohort. However, the high rate of logistical dropouts emphasizes the need to simplify screening processes but ensuring adequate genetic counselling, including online consultations and flexible scheduling, to enhance accessibility. Consistent with previous research, the primary motivation for participation was the desire to prevent the birth of a child with a severe genetic disorder [15, 16].

It should be also noted that the cohort represents the catchment area of a single primary care site in a middle-class district of Barcelona; consequently, it may not be representative of the broader national population. This introduces potential selection bias—particularly regarding education and socioeconomic status—thereby limiting the generalizability of the findings.

**Fig. 4:**
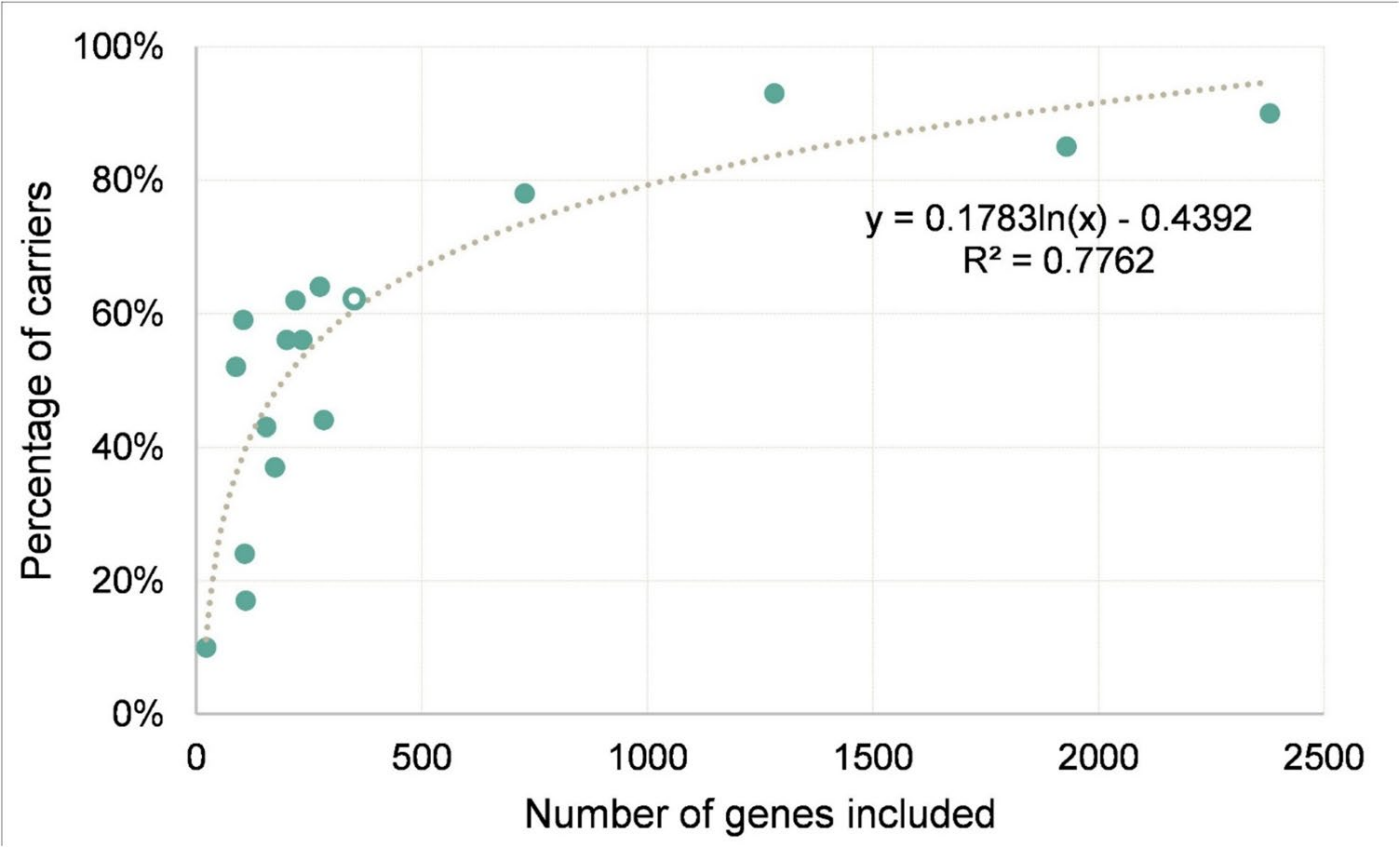
Scatter plot illustrating the percentage of individuals identified as carriers in relation to the number of recessive conditions screened. Colored data points represent aggregated results from previously published studies. The result of this study is represented by an empty circle. The trend follows a logarithmic pattern. Updated from Rowe and Wright [41]. Detailed information and references for these studies are provided in Supplementary Table 5

In line with previous large-scale carrier screening studies (Supplementary Table 5), 62% of participants carried at least one pathogenic or likely pathogenic variant (Fig. 4). The mean observed burden of 1.17 pathogenic variants per individual (1.9 among carriers) was slightly lower than the estimated global average of two variants per person [5, 6, 40], potentially reflecting differences in panel size, population-specific allele frequencies, and/or variant classification criteria. The observed carrier rate and variant distribution align with expectations, as the genes with the highest carrier frequencies (*GJB2*, *CFTR*, *ABCA4*, *PAH*, and *SMN1*) have been reported among the most prevalent disease-causing variants in diverse populations (Supplementary Table 5).

Notably, 4% of couples were identified as high-risk, a slightly higher proportion than previously reported in large-scale carrier screening programs [6–12]. This discrepancy may stem from several factors, including our smaller cohort size, the inclusion of additional high-prevalence carrier genes (e.g., *GJB2* and *ABCA4*), and the reporting of variants associated with milder phenotypes. In the two *GJB2* couples, one partner carried a common mild allele (p.Met34Thr or p.Val37Ile). Even when present in trans with a severe variant, these alleles are typically associated with milder, often manageable forms of hearing loss. The implications of this variable expressivity were thoroughly discussed during the genetic counselling sessions to ensure the couple had all the necessary information to support informed decision-making regarding their reproductive future. The variant identified in *GLA* (p.Arg356Gln) was classified as likely pathogenic at the time of analysis. However, conflicting evidence, including reports of unaffected hemizygotes in population datasets, functional data demonstrating substantial residual enzyme activity, and inconsistencies across expert submissions, raised uncertainty regarding its clinical significance. Therefore, after some debate, this result was returned with caution, carefully highlighting the limits of current knowledge, and arranged specialist follow-up. After revising the reporting policy, this variant would not be reported in future carrier screening contexts unless more definitive evidence becomes available. Notably, none of the high-risk couples identified in our cohort reported a family history suggestive of the detected conditions.

Among carrier couples, 75% modified their reproductive plans, opting for prenatal diagnosis or PGT. The decision to pursue reproductive interventions likely reflects the perceived severity of the condition. The couple who chose not to avoid transmission were carriers of *GJB2*, associated with nonsyndromic hearing loss, a condition generally considered manageable. In contrast, two of the other three couples were carriers of more severe, life-limiting disorders such as cystic fibrosis and Smith-Lemli-Opitz syndrome. This suggests that couples facing more serious conditions are more inclined to prioritize risk mitigation [42]. This observation demonstrates the clinical utility of preconception CS in enabling informed reproductive autonomy, a key outcome emphasized in guidelines [13, 20]. These findings reinforce the value of preconception timing, which expands options including PGT, contrasting the limited choices available post-conception [14]. Our result is consistent with previous research demonstrating that a significant proportion of couples take preventive reproductive measures following a positive screening result [42–44]. However, the small sample size of six identified high-risk couples limits the generalization of these findings, highlighting the need for larger studies within the population to validate these trends. Future research should explore how socioeconomic, cultural, and legal factors influence reproductive decision-making among at-risk couples and assess the long-term impact of carrier screening on birth outcomes and healthcare costs.

The identification of a female carrier of the X-linked *GLA* variant (Fabry disease; OMIM *300,644) in this carrier screening program illustrates the implications of incidental findings. While the primary goal of carrier screening is to assess reproductive risk, heterozygosity for *GLA* may also confer personal health implications. Due to skewed X-inactivation, female carriers can develop late-onset manifestations such as cardiomyopathy, renal dysfunction, or neuropathic pain, requiring multidisciplinary follow-up. In this case, the variant itself has been associated with a mild phenotype with late onset and reduced penetrance. Notably, the participant’s uncle was also found to be a carrier and was referred for specialized clinical evaluation. Another incidental finding was a carrier of *ATM*, a gene associated with ataxia telangiectasia and cancer predisposition. This result revealed actionable health information beyond reproductive counselling, challenging expanding implications of carrier screening [22, 45]. Importantly, the possibility of identifying such medically relevant incidental findings—including adult-onset conditions—had been pre-emptively addressed during the pre-test counselling sessions. Consequently, participants were fully informed and had consented to the reporting of these findings prior to testing. These cases highlight the ethical and logistical complexities of informed consent and variant disclosure, particularly for conditions with variable penetrance or unclear clinical actionability. Standardized protocols to unify reporting criteria, optimize informed consent, and engage specialist collaboration while ensuring risk management without overmedicalization are key, particularly in public health systems with limited resources.

The negative correlation between anxiety and perceived personal control observed in this study aligns with psychological models suggesting that greater perceived control can mitigate stress responses to uncertain situations, while knowledge can enhance autonomy [14]. Although 53% of participants reported reduced anxiety after testing, the significant drop in post-test survey responses (23%) induces possible response bias, as some participants may have disengaged from the study after receiving their results. This is consistent with previous research indicating that initial anxiety typically diminishes over time [46] but highlights the difficulty of assessing psychosocial outcomes in studies with low participation. Additionally, the significant association between education level and scientific knowledge reinforces the importance of personalized genetic counselling. While expanded carrier screening can empower informed reproductive decisions, its complexity, particularly with multi-condition panels, risks information overload [47]. These results suggest that screening programs should include exhaustive pre-test counselling and clear result explanations, particularly in public health systems where equitable access is still a major challenge [45]. Furthermore, implementing genetic education campaigns could help address possible misconceptions.

The sequential screening strategy proved to be effective in minimizing unnecessary male testing as previously reported [48]; however, further optimization is required before its scalability on a community level. One potential refinement would involve reducing the number of genetic counselling visits to two, attended by both partners. During the initial visit, blood samples could be collected from both individuals, but the male partner’s sample would only be analyzed if the female was identified as a carrier. This approach would eliminate the need for a separate disclosure visit for female results, optimizing the process so that the post-test visit focuses on delivering both partners’ results (if applicable) and discussing the couple’s reproductive risk. Future protocol enhancements could incorporate mandatory online educational modules coupled with brief comprehension assessments to streamline pre-test education. Implementing group counselling sessions for the pre-test phase could further optimize clinical resources by reducing the volume of individual appointments. However, tailored genetic counselling would remain accessible upon request and continue to be the standard of care for the disclosure of positive results.

Internationally, discussions focus on the optimal setting for carrier screening—whether within primary care or delivered through specialized genetics services—and on which and how many conditions to include. Given the limited overlap among existing gene panels, recent proposals advocate for selecting genes based on clinical utility rather than disease severity, which means to include those genes and variants for which decisions and actions that are personal in nature can be made [49]. Implementation models vary from couple‑based to individual screening, each with distinct logistical and economic considerations. Other ethical dilemmas include the appropriate mode of genetic counselling, the potential for stigmatization, the psychosocial impact of uncertain or incidental findings, and the risk of exacerbating health disparities. These ethical challenges highlight the need for careful consideration in their design and implementation.

The exclusion of CS from public healthcare systems exacerbates inequities, as access remains dependent on private resources [26]. While this study demonstrates the feasibility of public integration, several challenges should be addressed for broader implementation. First, logistical constraints including the need to scale genetic counselling and reproductive medicine capacity remain a critical barrier. Second, the small cohort size and recruitment from a single primary health care center in Barcelona may not represent demographic diversity and introduce selection bias. Furthermore, the low post-test survey response rate limits psychosocial conclusions, highlighting the need for longitudinal analyses with higher engagement. Additionally, although most high‑risk couples in this cohort altered their reproductive plans, larger studies are required to help understand the influence of socioeconomic, cultural, and health-related factors on reproductive decision-making in more diverse populations. Third, the gene panel, designed before updated guidelines [20–22] and key publications [23], requires further curation to align with current evidence and the population diversity and to ensure more consistent screening strategies. Gene panels, including CS panels, must remain dynamic and undergo regular updates as new pathogenicity evidence, population-specific risk data, therapeutic advancements, and guidelines from professional societies become available [50]. Finally, comprehensive cost-effectiveness studies are necessary to support the expansion of CS within public healthcare systems.

This study provides evidence for the feasibility and clinical value of integrating CS into Spain’s public healthcare system. By identifying at-risk couples and empowering informed reproductive choices, CS aligns with international recommendations for reducing the burden of severe genetic disorders [13, 17–22]. The program’s success in detecting actionable risks from AR and XLR conditions underscores its potential to address equity gaps in Spain’s healthcare system. Some of the limitations of the study, including a small cohort and single-center recruitment, highlight the necessity of more extensive trials with longitudinal follow-up to assess psychosocial outcomes, reproductive choices, and long-term cost-effectiveness.

## Supplementary Information

Below is the link to the electronic supplementary material.

ESM 1 PDF (269 KB)

ESM 2 XLSX (35.4 KB)

## Data Availability

Pathogenic variant data and survey answers have been submitted to GitHub (https:/github.com/GeneticaSantPau/Paper_Carriers). Full genetic data are not publicly available to protect the privacy of participants.
