# Supplementary material for: Integrating preconception carrier screening into public health: lessons learned from a pilot implementation study
Source: J Assist Reprod Genet. 2026 May 4;43(6):1711–22. doi: 10.1007/s10815-026-03871-9 (PMC13319850; doi:10.1007/s10815-026-03871-9)
Supplement: Supplementary file 1 — PDF (269 KB) [file 10815_2026_3871_MOESM1_ESM.pdf]

Supplementary table 3. *Demographic characteristics and response rates of study participants. Data are presented as frequency counts with percentages in parentheses.*

**Cohort**		**Women**	**Men**	**Total**
**Age**	**18-25**	9 (7%)	2 (6%)	11 (7%)
	**26-30**	42 (33%)	5 (16%)	47 (30%)
	**31-34**	36 (28%)	9 (29%)	45 (28%)
	**35-38**	40 (31%)	8 (26%)	48 (30%)
	**>38**	-	7 (23%)	7 (4%)
**Place of residence**	**Barcelona**	111 (86%)	25 (81%)	136 (85%)
	**Province of Barcelona**	12 (9%)	3 (9%)	15 (10%)
	**Catalonia**	4 (3%)	1 (3%)	5 (3%)
	**Abroad**	2 (2%)	1 (3%)	4 (3%)
**Level of education (ISCED classification)**	**2 (lower secondary education)**	2 (2%)	1 (3%)	3 (2%)
	**3 (upper secondary education)**	8 (6%)	6 (17%)	14 (9%)
	**4 (post-secondary non-tertiary education)**	5 (4%)	4 (11%)	9 (5%)
	**6 (Bachelor's or equivalent)**	39 (30%)	8 (23%)	47 (29%)
	**7 (Master's or equivalent)**	71 (55%)	12 (34%)	83 (51%)
	**8 (Doctoral or equivalent)**	4 (3%)	4 (11%)	8 (5%)
**Participation**	**Pre-test survey**	129 (85%)	31 (49%)	160 (74%)
	**Post-test survey**	36 (24%)	14 (22%)	50 (23%)

Supplementary table 4*. Pathogenic variants identified in carrier couples.*

Couple	Gene	Variant 1	Class	Variant 2	Class
1	*GJB2*	NM_004004.6:c.109G>A	Pathogenic (PP1$_{Strong}$, PS4$_{Moderate}$, PM1, PM2, PM5, PP2, PP3)	NM_004004.6:c.35delG	Pathogenic (PVS1$_{Strong}$, PS4, PM2, PM3, PP5)
2	*GJB2*	NM_004004.6.6:c.101T>C	Pathogenic (PM3$_{Very\ strong}$, PS4$_{Moderate}$, PM1, PM2, PM5, PP1, PP2, PP3)	NM_004004.6:c.35delG	Pathogenic (PVS1$_{Strong}$, PS4, PM2, PM3, PP5)
3	*CFTR*	NM_000492.4:c.1520_1522del	Pathogenic (PS1$_{Moderate}$, PM1, PM2, PM3, PM4, PM5, PP5)	NM_000492.4:c.1520_1522del	Pathogenic (PS1$_{Moderate}$, PM1, PM2, PM3, PM4, PM5, PP5)
4	*ABCA4*	NM_000350.3:c.6089G>A	Pathogenic (PM3$_{Very\ strong}$, PM1, PM2, PP2, PP3)	NM_000350.3:c.3113C>T	Likely pathogenic (PM1, PM2, PP2, PP5)
5	*DHCR7*	NM_001360.3:c.964-1G>C	Pathogenic (PM3$_{Very\ strong}$, PVS1$_{Strong}$, PM2, PS3$_{Supporting}$, PP1)	NM_001360.3:c.906C>G	Pathogenic (PM3$_{Very\ strong}$, PM1, PM2, PM5, PS3$_{Supporting}$, PP1, PP2, PP3)
6	*GLA*	NM_000169.3:c.1067G>A	Likely pathogenic (PS4$_{Moderate}$, PM5, PS3$_{Supporting}$, PP2)	-	-

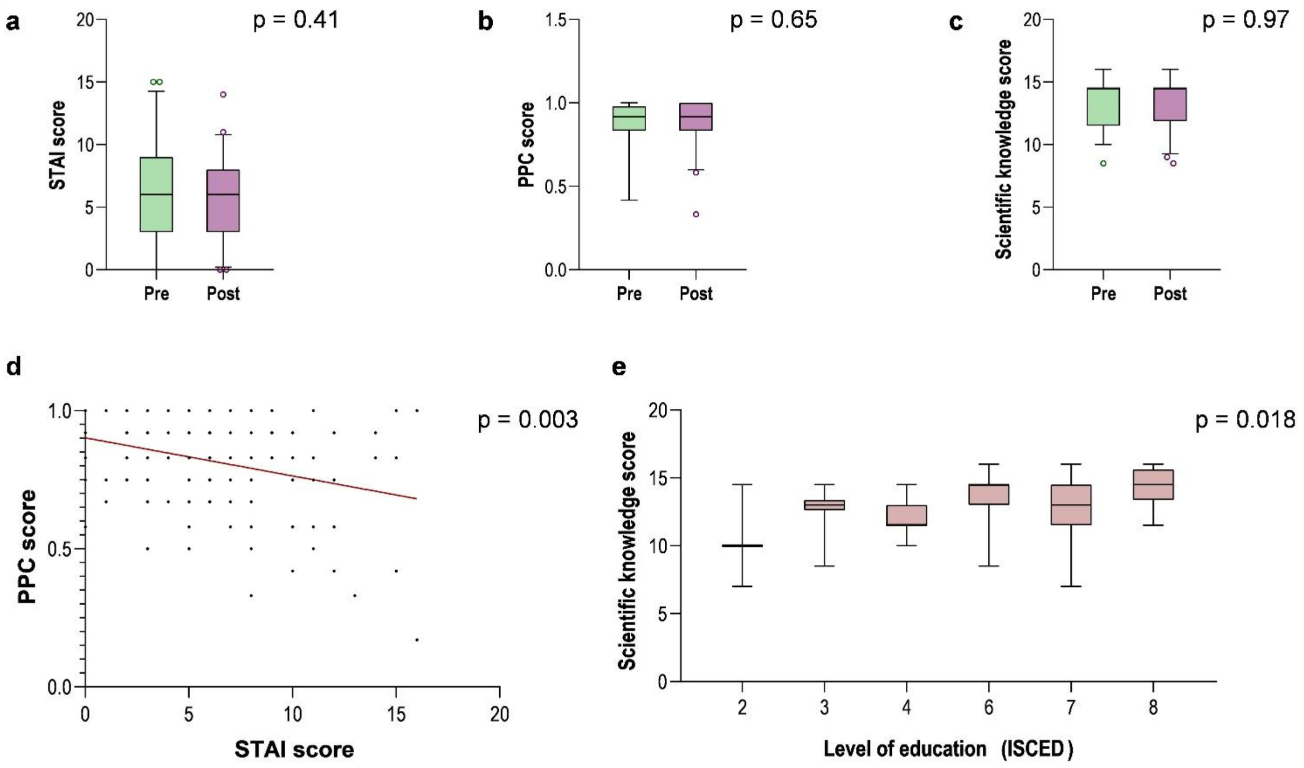

Supplementary figure. Surveys outcomes. **a-c**) Comparison of pre-test and post-test scores for STAI, PPC and scientific knowledge. Boxplots display group distributions, with whiskers representing the 5th–95th percentiles and outliers marked as circles (pre-test) and squares (post-test). Median values are shown as central lines, and boxes span the lower to upper quartiles. No significant differences were observed between pre- and post-test scores across all measures. **d**) Scatter plot showing the negative correlation between STAI and PPC scores (Spearman's correlation coefficient: Rho = -0.232). Higher anxiety levels are associated with lower perceived control. **e**) Association between scientific knowledge and education level. Boxplots illustrate the distribution of scientific knowledge scores across education levels (ISCED classification 3–9), with medians (central lines), interquartile ranges (boxes), and whiskers extending to minimum and maximum values. Higher education levels are associated with progressively greater scientific knowledge. One-way ANOVA (Kruskal-Wallis test) confirmed a statistically significant relationship.
